# 

**Published:** 1889-10

**Authors:** 


### BOOKS AND PAMPHLETS RECEIVED.

Hog Cholera, its history, nature and treatment, as determined by the inquiries and investigations of the Bureau of Animal Industry. 800 pp. 193. 14 colored plates. Washington, 1889.

Report of the U. S. Board of Inquiry concerning Epizootic diseases among swine. Washington, 1889.

Report upon Tuberculosis and its prevalence among the neat cattle of Rhode Island. By Charles H. Fisher, M.D., Sec'y State Board of Health. Providence, 1889.

Bulletin No. 5. Hatch Experiment Station, Massachusetts Agricultural College, Division of Entomology.

Considerations concerning some External Sources of Infection in their bearing on Preventive Medicine. By W. H. Welch, M.D. Reprinted from *Medical News.*

Secondary Mixed Infection in some of the Acute Infectious Diseases of Children. By Bayard Holmes, M.D. Reprinted from North American Practitioner.

Blindness and the Blind. By L. Webster Fox, M.D. Reprinted from Journal of Franklin Institute.

Accumulators and their Medical Use. By Robert Newman, M.D. Reprinted from *Philadelphia Med. Times.*

Zur Kenntness der Infections—Krankheiten niederer Thiere und Pflanzen von Dr. Wilhelm Zopf M.A.N. Bd. LII. No. 7. Nova acta der Ksl. Leop-Carol Deut Akad.

Beschreibung dreier Mikrocephalen-Gehirne nebst Vorstudien zur Anatomie der Mikrocephalie. Ab. 1 Von Dr. Felix Marchand, M.A.N. Bd. LIII. No. 3. Nova acta der Ksl. Leop-Carol Deut Akad.

Observations upon the Development of the Skull in *Neotoma fuscipes.* By R. W. Shufeldt, C.M.Z.S. Reprinted from Proceedings Academy of Natural Sciences.

Observations upon the Osteology of the Orders Tubenares and Steganopodes. By R. W. Shufeldt, U. S. A. Reprinted from Proceedings of U. S. Nat. Museum, Vol. XI.

Contributions to the Comparative Osteology of Arctic and Sub-Arctic Water Birds. Pt. IV. By R. W. Shufeldt, C.M.Z.S. Reprinted from Journal Anatomy and Physiology, Vol. XXIII.

Sixth Annual Report of the Bureau of Ethnology. By J. W. Powell, Director. Washington, 1888.

Annual Report of the Board of Regents of the Smithsonian Institution. Pt. 1. Washington, 1889.

Report of the Ornithologist and Mammalogist for the year 1888, Departmen of Agriculture. By C. Hart Merriam, M.D. Washington, 1889.

The English Sparrow in North America, especially in its relations to Agriculture. By Walter B. Barrows. Washington, 1889.

Report of the Commissioners of Fisheries of the State of New York. Albany, 1889.

New York Post-Graduate Medical School and Hospital, Eighth Annual Announcement, 1889–90.

New York College of Veterinary Surgeons and School of Comparative Medicine. Annual Announcement, 1889–90.

Kansas City University Medical College. Ninth Annual Announcement.

Baltimore College of Physicians and Surgeons. Annual Announcement, 1889–90.

Chicago Hahnemann Medical College and Hospital. Thirtieth Annual Announcement, 1889–90.

Annual Report of the President to the Corporation of Brown University.

ALBANY.—*Medical Annals.*

AUGSBURG.—*Wochenschrift fur Thierheilkunde und Viehzucht.*

AUGUSTA.—*The Sanitary Inspector.*

BALTIMORE.—*Johns Hopkins University Circulars.*

BATAVIA.—*Veeartsenijkundige Baden voor Nederlandsch Indie. Natuurkundig Tydschrift voor Nederlandsch Indie.*

BERLIN.—*Archiv fur Wissenschaftliche und Praktische Thierheilkunde.*

BOMBAY.—*Journal of the Natural History Society.* Vol. 4, No. 1.

BONN.—*Verhandlungen des Naturhistorischen Vereines.*

BRISBANE.—*Proceedings of the Royal Society of Queensland.* Vol. 6, Pt. 2–4. *Annual Meeting of the Royal Society.*

BRUSSELS.—*Bulletin de l' Academie Royale de Medecine.*

BUENOS AIRES.—*Anales de la Sociedad Cientifica Argentina.*

BUFFALO.—*Horse World.*

CAP ROUGE.—*Le Naturaliste Canadien.*

CHICAGO.—*Weekly Drovers' Journal.*

CINCINNATI.—*Journal of the Society of Natural History.*

CONCORD.—*The Sanitary Volunteer.*

COPENHAGEN.—*Tidsskrift for Veterinærer.*

DENVER.—*Proceedings Colorado Scientific Society.* Vol. 3, Pt. 1.

DETROIT.—*The Microscope.—The Pharmaceutical Era.*

DRESDEN.—*Jahresbericht der Gesellschaft fur Natur und Heilkunde Bericht des Vorstandes vom Aktien Verein Zoologescher Garten, 1888.*

EDINBURGH.—*Journal of Comparative Pathology and Therapeutics.*

FRANKFORT.—*Der Zoologische Garten.*

GHENT.—*Annales et Bulletin de la Societe de Medecine.*

GIESSEN.—*Bericht der Oberhessischen Gesellschaft fur Natur und Heilkunde, 1889.*

HAGUE.—*Verslag K. Zool. Bot. Genootschap,* 1888.

HALLE.—*Leopoldina, 1888.*

HARLEM.—*Archives Neerlandaises des Sciences exactes et Naturelles.* T. xxiii, Liv. 3–4.

HAVANA.—*Revista de Ciencias Medicas.*

JENA.—*Jenaische Zeitschrift fur Naturwissenschaft von der Medizinish Naturwissenschaftlichen Gesellschaft.*

KARLSRUHE.—*Thierarztliche Mittheilungen.*

KNOXVILLE.—*Agricultural Science.*

LAUSANNE.—*Bulletin de la Societe Vaudoise des Sciences Naturelles.* Nos. 97–98.

LONDON.—*The Veterinary Journal—The Veterinarian.*

LYON.—*Journal de Medecine Veterinaire et de Zootechnie.*

MADRAS.—*Quarterly Journal of Veterinary Science in India.*

MADRID.—*Gaceta Medico-Veterinaria.*

MELBOURNE.—*Annual Report of the Zoological and Acclimatization Society of Victoria,* 1889.

MILAN.—*La Clinica Veterinaria.*

MONTREAL.—*Canadian Record of Science.* Vol. iii, No. 7.

MULHOUSE.—*Bulletin de la Societe Industrielle.*

NEWPORT.—*Proceedings of the Natural History Society,* 1887–8.

NEW YORK.—*Medical Analectic—New York Medical Journal—Scientific American.—Forest and Stream.—American Veterinary Review.—Spirit of the Times.—The Sanitarian.—American Naturalist.*

OTTAWA.—*The Ottawa Naturalist.*

PALERMO.—*Il Naturalista Siciliano.*

PARIS.—*Le Progress Medical.—Recueil de Medicine Veterinaire.—Bulletin de la Societe Zoologique.* T. xiv, Nos. 5–6.

PAVIA.—*Bollettino Scientifico.*

PHILADELPHIA.—*Medical and Surgical Reporter.—Therapeutic Gazette.*

PISA.—*Giornale di Anat. Fisiol. e Patol. degli Animali.*

RED WING.—*Public Health in Minnesota.*

SHANGHAI.—*Journal of the China Branch of the Royal Asiatic Society.* Vol. xxiii, No. 2.

SOLEURE.—*Verhandlungen der Schweizerischen Naturforschenden Gesellschaft.*

STOCKHOLM.—*Tidskrift for Veterinar Med*[illegible]

ST. PETERSBURGH.—*Archives of Veteinary Scien*[illegible]

STUTTGART.—*Repertorium der Teirheilkunde.*

SYDNEY.—*Journal and Proceedings of the Royal Society of New South Wales.* Vol. xxii, Part 2. "*Proceedings of the Linnean Society of New South Wales.*" Vol. iii, Part 4. "*Act of Incorporation, Rules, List of Members, &c., of the Linnean Society.*" "*Annual Report of the New South Wales Zoological Society, 1889.*"

TOULOUSE.—*Revue Veterinaire.*

TURIN.—*Bollettino diMusee de Zoologia ed Anatomi Comparata.*

UNDINE.—*Giornale di Veterinaria Militare.*

VALENCIA.—*La Cronica Medica.*

VIENNA.—*Œstcrreichische Monatschrift fur Thierheilkunde. Ornis. Verhandlungen der K. K Zoologisch-botanischen Gesellschaft.* Bd. xxxix, 1–2. *Monatschrift des vereins der Thierartze in Œsterreich.*

WILMINGTON.—*Delaware Farm aud Home.*

WURZBRUG.—*Sittzungsberichte der Physikalisch-Medicinschen Gesellschaft.*

ZURICH.—*Schweizer Achiv fur Thierheilkunde.*

